# (1*S*,3*R*)-3-Isobutyl-2,3-dihydro­spiro[benzo[*f*]isoindole-1,3′-indoline]-2′,4,9-trione methanol monosolvate

**DOI:** 10.1107/S1600536812032643

**Published:** 2012-07-25

**Authors:** Garima Sharma, S. Vasanth Kumar, Habibah A. Wahab, Mohd Mustaqim Rosli, Hoong-Kun Fun

**Affiliations:** a Department of Chemistry, Karunya University, Coimbatore, India; b School of Pharmaceutical Sciences, Universiti Sains Malaysia, 11800 USM, Penang, Malaysia; c Malaysian Institute of Pharmaceuticals and Nutraceuticals, Ministry of Science, Technology and Innovation, Halaman Bukit Gambir, 11700 Bayan Lepas, Penang, Malaysia; d X-ray Crystallography Unit, School of Physics, Universiti Sains Malaysia, 11800 USM, Penang, Malaysia

## Abstract

In the title compound, C_23_H_20_N_2_O_3_·CH_3_OH, the hexa­hydro-1*H*-benzo[*f*]isoindole and indoline rings are planar, with maximum deviations of 0.092 (1) and −0.095 (1) Å, respectively. The dihedral angle between these two rings is 88.03 (4)°. An O—H⋯N inter­action links the main mol­ecule and the methanol solvent mol­ecule. An intra­molecular C—H⋯O inter­action forms an *S*(6) ring motif. In the crystal, the mol­ecules form two-dimensional layers parallel to the *bc* plane through N—H⋯O and C—H⋯O inter­actions.

## Related literature

For biological activities of naphtho­quinones, see: Babula *et al.* (2007 ▶). For detailed literature on naphtho­quinone chemistry, see: Chen *et al.* (2011 ▶); Silva *et al.* (2002 ▶). For hydrogen-bond motifs, see: Bernstein *et al.* (1995 ▶). For the stability of the temperature controller used for the data collection, see: Cosier & Glazer (1986 ▶).
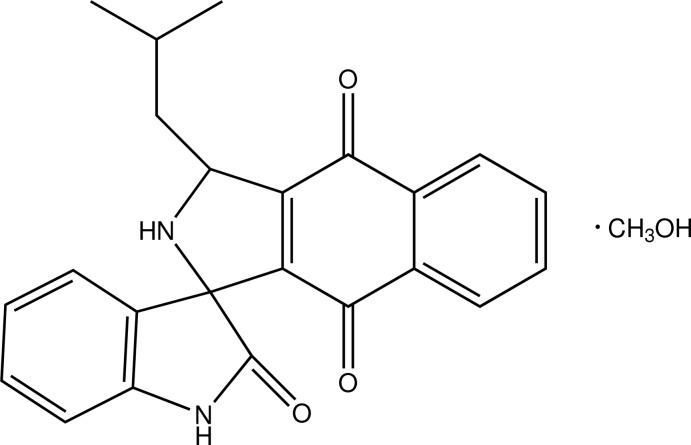


## Experimental

### 

#### Crystal data

C_23_H_20_N_2_O_3_·CH_4_O
*M* *_r_* = 404.45
Monoclinic, 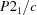

*a* = 10.8485 (2) Å
*b* = 11.9605 (2) Å
*c* = 16.5705 (3) Å
β = 111.246 (1)°
*V* = 2003.95 (6) Å^3^
*Z* = 4
Mo *K*α radiation
μ = 0.09 mm^−1^
*T* = 100 K
0.32 × 0.20 × 0.11 mm

#### Data collection

Bruker SMART APEXII CCD area-detector diffractometer
Absorption correction: multi-scan (*SADABS*; Bruker, 2009 ▶) *T*_min_ = 0.971, *T*_max_ = 0.990
23197 measured reflections
5871 independent reflections
4460 reflections with *I* > 2σ(*I*)
*R*_int_ = 0.035

#### Refinement

*R*[*F* ^2^ > 2σ(*F* ^2^)] = 0.053
*wR*(*F* ^2^) = 0.135
*S* = 1.03
5871 reflections
286 parameters
H atoms treated by a mixture of independent and constrained refinement
Δρ_max_ = 0.63 e Å^−3^
Δρ_min_ = −0.46 e Å^−3^

### 

Data collection: *APEX2* (Bruker, 2009 ▶); cell refinement: *SAINT* (Bruker, 2009 ▶); data reduction: *SAINT*; program(s) used to solve structure: *SHELXTL* (Sheldrick, 2008 ▶); program(s) used to refine structure: *SHELXTL*; molecular graphics: *SHELXTL*; software used to prepare material for publication: *SHELXTL* and *PLATON* (Spek, 2009 ▶).

## Supplementary Material

Crystal structure: contains datablock(s) I, global. DOI: 10.1107/S1600536812032643/xu5595sup1.cif

Structure factors: contains datablock(s) I. DOI: 10.1107/S1600536812032643/xu5595Isup2.hkl

Supplementary material file. DOI: 10.1107/S1600536812032643/xu5595Isup3.cml

Additional supplementary materials:  crystallographic information; 3D view; checkCIF report

## Figures and Tables

**Table 1:** Hydrogen-bond geometry (Å, °)

*D*—H⋯*A*	*D*—H	H⋯*A*	*D*⋯*A*	*D*—H⋯*A*
N1—H1N1⋯O2^i^	0.903 (19)	2.249 (19)	3.1410 (16)	169.7 (17)
N2—H1N2⋯O4^ii^	0.89 (2)	1.97 (2)	2.8346 (18)	165 (2)
O4—H1O4⋯N1	0.93 (3)	1.88 (3)	2.8085 (18)	174 (2)
C13—H13*B*⋯O1	0.99	2.56	3.1919 (18)	121
C19—H19*A*⋯O3^i^	0.95	2.57	3.3368 (18)	138

Symmetry codes: (i) 
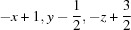
; (ii) 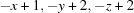.

**Fig. 1.**
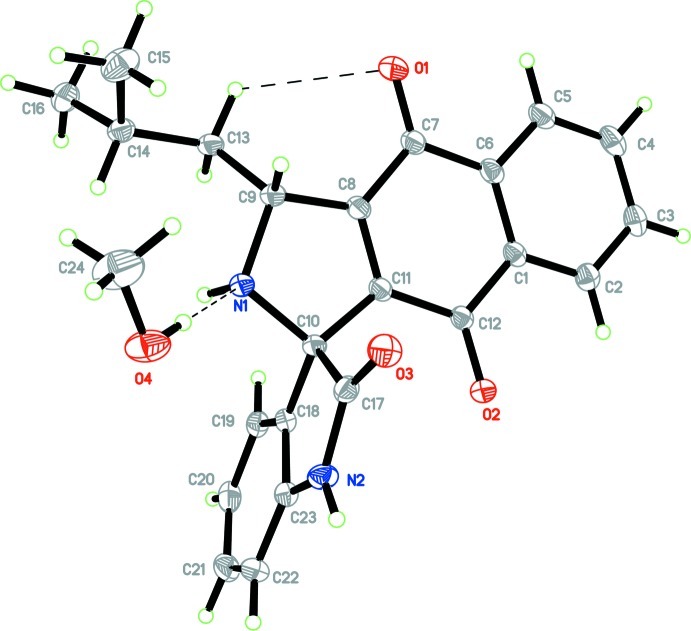
The molecular structure, showing 50% probability displacement ellipsoids. Hydrogen atoms are shown as spheres of arbitrary radius.

**Fig. 2.**
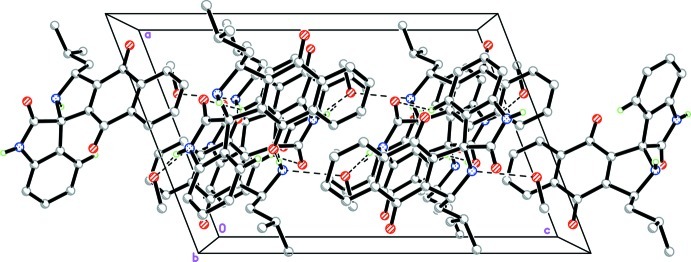
The crystal packing of (I). Dashed lines indicate hydrogen bonds. H atoms not involved in the hydrogen bond interactions have been omitted for clarity.

## supplementary crystallographic information

### Comment

Naphthoquinones are known to possess various biological activities such as cyto-toxicity as well as antibacterial, antifungal, antiviral, insecticidal, anti-inflammatory, and antipyretic (Babula *et al.*, 2007) properties. Recently, there have been a few efforts to conduct 1,3-cycloaddition involving naphthoquinones (Chen *et al.*, 2011; Silva *et al.*, 2002).

In the title compound, Fig. 1, the hexahydro-1*H*-benzo[*f*]isoindole (N1/C1–C12) and indoline (N2/C10/C17–C23) rings are planar with the maximum deviations of 0.092 (1) Å from atom N1 and -0.095 (1) Å from atom C10. The two rings make a dihedral angle of 88.03 (4)°. An O4—H1O4···N1 interaction links the main molecule with the methanol solvent molecule. An intramolecular interaction of C13—H13B···O1 forms an S(6) ring motif (Fig. 1).

In the crystal, the molecules form two-dimensional layers parallel to the *bc*-plane through the intermolecular interactions of N1—H1N1···O2^i^, N2—H1N2···O4^ii^ and C19—H19A···O3^i^ (Fig. 2).

### Experimental

A mixture of isatin (0.147 g, 1 mmol), *L*-leucine (0.131 g, 1 mmol) and 1,4-napthoquinone (0.158 g, 1 mmol) was refluxed in methanol (6 ml) until the disappearance of the starting material (monitored by thin layer chromatography, TLC). After standing for 1 h, the product of the reaction mixture was washed with cool water (2 × 25 ml) and cool ethanol (2 × 0.5 ml). The crude product was recrystallized from appropriate solvent to afford pure product (90% yield).

### Refinement

N-bound H atoms were located from a difference Fourier map and freely refined. The remaining H atoms were positioned geometrically and refined using a riding model with C—H = 0.95–1.00 Å and *U*_iso_(H) = 1.2*U*_eq_(C) or 1.5*U*_eq_(C) for methyl H atoms. A rotating group model was applied to the methyl group.

### Crystal data

**Table:**

C_23_H_20_N_2_O_3_·CH_4_O	*F*(000) = 856
*M**_r_* = 404.45	*D*_x_ = 1.341 Mg m^−^^3^
Monoclinic, *P*2_1_/*c*	Mo *K*α radiation, λ = 0.71073 Å
Hall symbol: -P 2ybc	Cell parameters from 7290 reflections
*a* = 10.8485 (2) Å	θ = 2.6–30.1°
*b* = 11.9605 (2) Å	µ = 0.09 mm^−^^1^
*c* = 16.5705 (3) Å	*T* = 100 K
β = 111.246 (1)°	Block, brown
*V* = 2003.95 (6) Å^3^	0.32 × 0.20 × 0.11 mm
*Z* = 4	

### Data collection

**Table:**

Bruker SMART APEXII CCD area-detector diffractometer	5871 independent reflections
Radiation source: fine-focus sealed tube	4460 reflections with *I* > 2σ(*I*)
Graphite monochromator	*R*_int_ = 0.035
φ and ω scans	θ_max_ = 30.1°, θ_min_ = 2.0°
Absorption correction: multi-scan (*SADABS*; Bruker, 2009)	*h* = −15→14
*T*_min_ = 0.971, *T*_max_ = 0.990	*k* = −14→16
23197 measured reflections	*l* = −23→23

### Refinement

**Table:**

Refinement on *F*^2^	Primary atom site location: structure-invariant direct methods
Least-squares matrix: full	Secondary atom site location: difference Fourier map
*R*[*F*^2^ > 2σ(*F*^2^)] = 0.053	Hydrogen site location: inferred from neighbouring sites
*wR*(*F*^2^) = 0.135	H atoms treated by a mixture of independent and constrained refinement
*S* = 1.03	*w* = 1/[σ^2^(*F*_o_^2^) + (0.0597*P*)^2^ + 0.9585*P*] where *P* = (*F*_o_^2^ + 2*F*_c_^2^)/3
5871 reflections	(Δ/σ)_max_ < 0.001
286 parameters	Δρ_max_ = 0.63 e Å^−^^3^
0 restraints	Δρ_min_ = −0.46 e Å^−^^3^

### Special details

**Table:**

Experimental. The crystal was placed in the cold stream of an Oxford Cryosystems Cobra open-flow nitrogen cryostat (Cosier & Glazer, 1986) operating at 100.0 (1) K.
Geometry. All e.s.d.'s (except the e.s.d. in the dihedral angle between two l.s. planes) are estimated using the full covariance matrix. The cell e.s.d.'s are taken into account individually in the estimation of e.s.d.'s in distances, angles and torsion angles; correlations between e.s.d.'s in cell parameters are only used when they are defined by crystal symmetry. An approximate (isotropic) treatment of cell e.s.d.'s is used for estimating e.s.d.'s involving l.s. planes.
Refinement. Refinement of *F*^2^ against ALL reflections. The weighted *R*-factor *wR* and goodness of fit *S* are based on *F*^2^, conventional *R*-factors *R* are based on *F*, with *F* set to zero for negative *F*^2^. The threshold expression of *F*^2^ > σ(*F*^2^) is used only for calculating *R*-factors(gt) *etc*. and is not relevant to the choice of reflections for refinement. *R*-factors based on *F*^2^ are statistically about twice as large as those based on *F*, and *R*-factors based on ALL data will be even larger.

### Fractional atomic coordinates and isotropic or equivalent isotropic displacement parameters (Å^2^)

**Table:**

	*x*	*y*	*z*	*U*_iso_*/*U*_eq_	
O1	0.09694 (11)	0.90322 (10)	0.51378 (7)	0.0300 (3)	
O2	0.56629 (10)	1.07980 (8)	0.70838 (7)	0.0186 (2)	
O3	0.35764 (11)	1.07705 (9)	0.83896 (7)	0.0235 (2)	
N1	0.33921 (12)	0.82889 (10)	0.78597 (8)	0.0160 (2)	
N2	0.56151 (12)	1.00095 (10)	0.91874 (8)	0.0187 (3)	
C1	0.40186 (14)	1.06328 (11)	0.56677 (9)	0.0159 (3)	
C2	0.46388 (15)	1.13840 (12)	0.52980 (9)	0.0187 (3)	
H2A	0.5469	1.1698	0.5640	0.022*	
C3	0.40491 (16)	1.16770 (12)	0.44316 (10)	0.0219 (3)	
H3A	0.4469	1.2198	0.4183	0.026*	
C4	0.28436 (16)	1.12071 (13)	0.39287 (10)	0.0234 (3)	
H4A	0.2450	1.1399	0.3334	0.028*	
C5	0.22110 (16)	1.04605 (13)	0.42882 (9)	0.0218 (3)	
H5A	0.1385	1.0146	0.3940	0.026*	
C6	0.27860 (14)	1.01691 (11)	0.51630 (9)	0.0179 (3)	
C7	0.20717 (15)	0.93985 (12)	0.55492 (9)	0.0198 (3)	
C8	0.27704 (14)	0.90981 (12)	0.64722 (9)	0.0172 (3)	
C9	0.22425 (14)	0.83749 (12)	0.70212 (9)	0.0170 (3)	
H9A	0.1503	0.8777	0.7122	0.020*	
C10	0.44298 (13)	0.91165 (11)	0.78621 (9)	0.0146 (3)	
C11	0.39672 (14)	0.95085 (11)	0.69362 (9)	0.0155 (3)	
C12	0.46506 (14)	1.03460 (11)	0.66010 (9)	0.0148 (3)	
C13	0.17636 (14)	0.72159 (12)	0.66517 (9)	0.0177 (3)	
H13A	0.2508	0.6815	0.6570	0.021*	
H13B	0.1061	0.7307	0.6074	0.021*	
C14	0.12248 (14)	0.64910 (12)	0.72118 (10)	0.0184 (3)	
H14A	0.1887	0.6484	0.7817	0.022*	
C15	−0.00825 (16)	0.69353 (14)	0.72296 (12)	0.0297 (4)	
H15A	−0.0398	0.6443	0.7587	0.044*	
H15B	−0.0738	0.6957	0.6639	0.044*	
H15C	0.0048	0.7691	0.7475	0.044*	
C16	0.10456 (16)	0.52942 (13)	0.68612 (11)	0.0238 (3)	
H16A	0.0629	0.4839	0.7183	0.036*	
H16B	0.1911	0.4977	0.6929	0.036*	
H16C	0.0482	0.5300	0.6246	0.036*	
C17	0.44567 (14)	1.00939 (12)	0.84967 (9)	0.0170 (3)	
C18	0.58027 (13)	0.86393 (11)	0.82701 (9)	0.0151 (3)	
C19	0.64232 (14)	0.77902 (12)	0.79995 (9)	0.0182 (3)	
H19A	0.6009	0.7448	0.7450	0.022*	
C20	0.76752 (15)	0.74465 (13)	0.85537 (10)	0.0223 (3)	
H20A	0.8126	0.6872	0.8376	0.027*	
C21	0.82649 (15)	0.79390 (14)	0.93626 (11)	0.0252 (3)	
H21A	0.9106	0.7680	0.9737	0.030*	
C22	0.76496 (15)	0.88039 (13)	0.96365 (10)	0.0232 (3)	
H22A	0.8056	0.9140	1.0189	0.028*	
C23	0.64231 (14)	0.91542 (12)	0.90724 (9)	0.0172 (3)	
O4	0.31230 (12)	0.84323 (11)	0.94796 (8)	0.0309 (3)	
C24	0.1798 (2)	0.8342 (2)	0.93687 (13)	0.0425 (5)	
H24A	0.1507	0.7567	0.9226	0.064*	
H24B	0.1274	0.8835	0.8896	0.064*	
H24C	0.1676	0.8561	0.9905	0.064*	
H1N1	0.3759 (17)	0.7609 (16)	0.7870 (11)	0.021 (4)*	
H1N2	0.587 (2)	1.0493 (17)	0.9621 (13)	0.031 (5)*	
H1O4	0.317 (2)	0.842 (2)	0.8928 (17)	0.056 (7)*	

### Atomic displacement parameters (Å^2^)

**Table:**

	*U*^11^	*U*^22^	*U*^33^	*U*^12^	*U*^13^	*U*^23^
O1	0.0248 (6)	0.0283 (6)	0.0245 (6)	−0.0059 (5)	−0.0059 (5)	0.0056 (5)
O2	0.0176 (5)	0.0176 (5)	0.0178 (5)	−0.0019 (4)	0.0029 (4)	−0.0001 (4)
O3	0.0241 (6)	0.0198 (5)	0.0255 (6)	0.0038 (4)	0.0076 (5)	−0.0032 (4)
N1	0.0158 (6)	0.0149 (6)	0.0149 (5)	−0.0019 (4)	0.0027 (5)	−0.0005 (4)
N2	0.0202 (6)	0.0187 (6)	0.0149 (6)	−0.0024 (5)	0.0036 (5)	−0.0056 (5)
C1	0.0193 (7)	0.0131 (6)	0.0137 (6)	0.0038 (5)	0.0042 (5)	−0.0008 (5)
C2	0.0237 (7)	0.0154 (7)	0.0175 (7)	0.0014 (5)	0.0078 (6)	−0.0017 (5)
C3	0.0307 (8)	0.0182 (7)	0.0188 (7)	0.0041 (6)	0.0114 (6)	0.0035 (5)
C4	0.0309 (8)	0.0223 (7)	0.0152 (7)	0.0071 (6)	0.0060 (6)	0.0032 (5)
C5	0.0246 (7)	0.0191 (7)	0.0163 (7)	0.0037 (6)	0.0009 (6)	−0.0003 (5)
C6	0.0212 (7)	0.0138 (6)	0.0155 (6)	0.0036 (5)	0.0028 (5)	−0.0003 (5)
C7	0.0207 (7)	0.0150 (6)	0.0176 (7)	0.0002 (5)	−0.0004 (6)	0.0002 (5)
C8	0.0180 (7)	0.0141 (6)	0.0157 (6)	0.0005 (5)	0.0018 (5)	0.0005 (5)
C9	0.0148 (6)	0.0164 (6)	0.0165 (6)	−0.0011 (5)	0.0017 (5)	−0.0002 (5)
C10	0.0151 (6)	0.0139 (6)	0.0126 (6)	−0.0014 (5)	0.0024 (5)	−0.0011 (5)
C11	0.0176 (6)	0.0136 (6)	0.0130 (6)	0.0012 (5)	0.0026 (5)	0.0000 (5)
C12	0.0166 (6)	0.0118 (6)	0.0145 (6)	0.0020 (5)	0.0040 (5)	−0.0006 (5)
C13	0.0155 (6)	0.0171 (7)	0.0168 (6)	−0.0019 (5)	0.0013 (5)	−0.0012 (5)
C14	0.0161 (6)	0.0182 (7)	0.0189 (7)	−0.0016 (5)	0.0039 (5)	−0.0015 (5)
C15	0.0235 (8)	0.0269 (8)	0.0410 (10)	−0.0014 (6)	0.0146 (7)	−0.0059 (7)
C16	0.0257 (8)	0.0176 (7)	0.0269 (8)	−0.0032 (6)	0.0080 (6)	−0.0015 (6)
C17	0.0194 (7)	0.0145 (6)	0.0172 (6)	−0.0024 (5)	0.0066 (6)	−0.0010 (5)
C18	0.0156 (6)	0.0142 (6)	0.0144 (6)	−0.0005 (5)	0.0043 (5)	0.0025 (5)
C19	0.0203 (7)	0.0163 (7)	0.0190 (7)	−0.0016 (5)	0.0085 (6)	0.0020 (5)
C20	0.0215 (7)	0.0187 (7)	0.0298 (8)	0.0030 (6)	0.0130 (6)	0.0069 (6)
C21	0.0166 (7)	0.0272 (8)	0.0285 (8)	0.0015 (6)	0.0043 (6)	0.0110 (6)
C22	0.0198 (7)	0.0275 (8)	0.0172 (7)	−0.0036 (6)	0.0004 (6)	0.0033 (6)
C23	0.0180 (7)	0.0174 (7)	0.0152 (6)	−0.0026 (5)	0.0048 (5)	0.0019 (5)
O4	0.0261 (6)	0.0436 (7)	0.0229 (6)	−0.0067 (5)	0.0087 (5)	−0.0121 (5)
C24	0.0354 (10)	0.0640 (14)	0.0313 (10)	−0.0139 (9)	0.0161 (8)	−0.0126 (9)

### Geometric parameters (Å, º)

**Table:**

O1—C7	1.2231 (18)	C11—C12	1.470 (2)
O2—C12	1.2254 (17)	C13—C14	1.532 (2)
O3—C17	1.2147 (18)	C13—H13A	0.9900
N1—C9	1.4969 (18)	C13—H13B	0.9900
N1—C10	1.4979 (18)	C14—C15	1.525 (2)
N1—H1N1	0.902 (19)	C14—C16	1.531 (2)
N2—C17	1.3627 (19)	C14—H14A	1.0000
N2—C23	1.4043 (19)	C15—H15A	0.9800
N2—H1N2	0.88 (2)	C15—H15B	0.9800
C1—C2	1.391 (2)	C15—H15C	0.9800
C1—C6	1.409 (2)	C16—H16A	0.9800
C1—C12	1.4870 (19)	C16—H16B	0.9800
C2—C3	1.388 (2)	C16—H16C	0.9800
C2—H2A	0.9500	C18—C19	1.380 (2)
C3—C4	1.390 (2)	C18—C23	1.3969 (19)
C3—H3A	0.9500	C19—C20	1.398 (2)
C4—C5	1.386 (2)	C19—H19A	0.9500
C4—H4A	0.9500	C20—C21	1.390 (2)
C5—C6	1.3989 (19)	C20—H20A	0.9500
C5—H5A	0.9500	C21—C22	1.393 (2)
C6—C7	1.490 (2)	C21—H21A	0.9500
C7—C8	1.484 (2)	C22—C23	1.385 (2)
C8—C11	1.3395 (19)	C22—H22A	0.9500
C8—C9	1.510 (2)	O4—C24	1.386 (2)
C9—C13	1.528 (2)	O4—H1O4	0.93 (3)
C9—H9A	1.0000	C24—H24A	0.9800
C10—C11	1.5059 (19)	C24—H24B	0.9800
C10—C18	1.5072 (19)	C24—H24C	0.9800
C10—C17	1.5656 (19)		
			
C9—N1—C10	109.25 (11)	C14—C13—H13A	108.6
C9—N1—H1N1	107.0 (11)	C9—C13—H13B	108.6
C10—N1—H1N1	105.6 (12)	C14—C13—H13B	108.6
C17—N2—C23	111.78 (12)	H13A—C13—H13B	107.5
C17—N2—H1N2	123.9 (13)	C15—C14—C16	110.01 (13)
C23—N2—H1N2	123.9 (13)	C15—C14—C13	112.09 (13)
C2—C1—C6	119.98 (13)	C16—C14—C13	108.85 (12)
C2—C1—C12	119.52 (13)	C15—C14—H14A	108.6
C6—C1—C12	120.48 (13)	C16—C14—H14A	108.6
C3—C2—C1	120.22 (14)	C13—C14—H14A	108.6
C3—C2—H2A	119.9	C14—C15—H15A	109.5
C1—C2—H2A	119.9	C14—C15—H15B	109.5
C2—C3—C4	119.93 (15)	H15A—C15—H15B	109.5
C2—C3—H3A	120.0	C14—C15—H15C	109.5
C4—C3—H3A	120.0	H15A—C15—H15C	109.5
C5—C4—C3	120.53 (14)	H15B—C15—H15C	109.5
C5—C4—H4A	119.7	C14—C16—H16A	109.5
C3—C4—H4A	119.7	C14—C16—H16B	109.5
C4—C5—C6	120.12 (14)	H16A—C16—H16B	109.5
C4—C5—H5A	119.9	C14—C16—H16C	109.5
C6—C5—H5A	119.9	H16A—C16—H16C	109.5
C5—C6—C1	119.20 (14)	H16B—C16—H16C	109.5
C5—C6—C7	119.56 (13)	O3—C17—N2	127.52 (13)
C1—C6—C7	121.22 (12)	O3—C17—C10	125.24 (13)
O1—C7—C8	121.25 (14)	N2—C17—C10	107.21 (12)
O1—C7—C6	122.55 (13)	C19—C18—C23	120.68 (13)
C8—C7—C6	116.20 (12)	C19—C18—C10	130.70 (13)
C11—C8—C7	121.98 (14)	C23—C18—C10	108.48 (12)
C11—C8—C9	111.37 (12)	C18—C19—C20	118.38 (14)
C7—C8—C9	126.57 (12)	C18—C19—H19A	120.8
N1—C9—C8	103.19 (11)	C20—C19—H19A	120.8
N1—C9—C13	110.95 (11)	C21—C20—C19	120.48 (15)
C8—C9—C13	115.22 (12)	C21—C20—H20A	119.8
N1—C9—H9A	109.1	C19—C20—H20A	119.8
C8—C9—H9A	109.1	C20—C21—C22	121.43 (14)
C13—C9—H9A	109.1	C20—C21—H21A	119.3
N1—C10—C11	103.32 (10)	C22—C21—H21A	119.3
N1—C10—C18	111.75 (11)	C23—C22—C21	117.46 (14)
C11—C10—C18	119.03 (12)	C23—C22—H22A	121.3
N1—C10—C17	109.04 (11)	C21—C22—H22A	121.3
C11—C10—C17	111.72 (11)	C22—C23—C18	121.51 (14)
C18—C10—C17	101.93 (11)	C22—C23—N2	128.54 (14)
C8—C11—C12	123.34 (12)	C18—C23—N2	109.95 (12)
C8—C11—C10	111.50 (13)	C24—O4—H1O4	106.8 (15)
C12—C11—C10	124.73 (12)	O4—C24—H24A	109.5
O2—C12—C11	120.62 (12)	O4—C24—H24B	109.5
O2—C12—C1	122.93 (13)	H24A—C24—H24B	109.5
C11—C12—C1	116.40 (12)	O4—C24—H24C	109.5
C9—C13—C14	114.79 (12)	H24A—C24—H24C	109.5
C9—C13—H13A	108.6	H24B—C24—H24C	109.5
			
C6—C1—C2—C3	−0.2 (2)	C10—C11—C12—O2	−1.2 (2)
C12—C1—C2—C3	−178.75 (13)	C8—C11—C12—C1	−7.1 (2)
C1—C2—C3—C4	−0.9 (2)	C10—C11—C12—C1	−178.98 (12)
C2—C3—C4—C5	1.1 (2)	C2—C1—C12—O2	5.0 (2)
C3—C4—C5—C6	−0.3 (2)	C6—C1—C12—O2	−173.59 (13)
C4—C5—C6—C1	−0.8 (2)	C2—C1—C12—C11	−177.33 (13)
C4—C5—C6—C7	177.73 (14)	C6—C1—C12—C11	4.10 (19)
C2—C1—C6—C5	1.0 (2)	N1—C9—C13—C14	63.48 (16)
C12—C1—C6—C5	179.55 (13)	C8—C9—C13—C14	−179.76 (12)
C2—C1—C6—C7	−177.48 (13)	C9—C13—C14—C15	69.24 (16)
C12—C1—C6—C7	1.1 (2)	C9—C13—C14—C16	−168.83 (12)
C5—C6—C7—O1	−2.5 (2)	C23—N2—C17—O3	−177.45 (15)
C1—C6—C7—O1	175.95 (15)	C23—N2—C17—C10	4.59 (16)
C5—C6—C7—C8	177.82 (13)	N1—C10—C17—O3	−67.24 (18)
C1—C6—C7—C8	−3.7 (2)	C11—C10—C17—O3	46.32 (19)
O1—C7—C8—C11	−178.74 (15)	C18—C10—C17—O3	174.49 (14)
C6—C7—C8—C11	0.9 (2)	N1—C10—C17—N2	110.78 (13)
O1—C7—C8—C9	−2.3 (2)	C11—C10—C17—N2	−135.65 (13)
C6—C7—C8—C9	177.34 (13)	C18—C10—C17—N2	−7.49 (14)
C10—N1—C9—C8	11.53 (14)	N1—C10—C18—C19	67.08 (19)
C10—N1—C9—C13	135.47 (12)	C11—C10—C18—C19	−53.3 (2)
C11—C8—C9—N1	−7.48 (16)	C17—C10—C18—C19	−176.60 (14)
C7—C8—C9—N1	175.79 (13)	N1—C10—C18—C23	−108.46 (13)
C11—C8—C9—C13	−128.56 (13)	C11—C10—C18—C23	131.21 (13)
C7—C8—C9—C13	54.71 (19)	C17—C10—C18—C23	7.86 (14)
C9—N1—C10—C11	−11.27 (14)	C23—C18—C19—C20	1.1 (2)
C9—N1—C10—C18	−140.42 (12)	C10—C18—C19—C20	−173.99 (14)
C9—N1—C10—C17	107.68 (12)	C18—C19—C20—C21	1.1 (2)
C7—C8—C11—C12	4.6 (2)	C19—C20—C21—C22	−1.7 (2)
C9—C8—C11—C12	−172.30 (13)	C20—C21—C22—C23	0.2 (2)
C7—C8—C11—C10	177.44 (13)	C21—C22—C23—C18	2.0 (2)
C9—C8—C11—C10	0.53 (17)	C21—C22—C23—N2	−179.06 (14)
N1—C10—C11—C8	6.65 (15)	C19—C18—C23—C22	−2.7 (2)
C18—C10—C11—C8	131.18 (13)	C10—C18—C23—C22	173.37 (13)
C17—C10—C11—C8	−110.43 (14)	C19—C18—C23—N2	178.20 (13)
N1—C10—C11—C12	179.36 (12)	C10—C18—C23—N2	−5.73 (16)
C18—C10—C11—C12	−56.11 (18)	C17—N2—C23—C22	−178.43 (15)
C17—C10—C11—C12	62.28 (18)	C17—N2—C23—C18	0.59 (17)
C8—C11—C12—O2	170.65 (14)		

### Hydrogen-bond geometry (Å, º)

**Table:**

*D*—H···*A*	*D*—H	H···*A*	*D*···*A*	*D*—H···*A*
N1—H1*N*1···O2^i^	0.903 (19)	2.249 (19)	3.1410 (16)	169.7 (17)
N2—H1*N*2···O4^ii^	0.89 (2)	1.97 (2)	2.8346 (18)	165 (2)
O4—H1*O*4···N1	0.93 (3)	1.88 (3)	2.8085 (18)	174 (2)
C13—H13*B*···O1	0.99	2.56	3.1919 (18)	121
C19—H19*A*···O3^i^	0.95	2.57	3.3368 (18)	138

Symmetry codes: (i) −*x*+1, *y*−1/2, −*z*+3/2; (ii) −*x*+1, −*y*+2, −*z*+2.
